# Complete Genome Sequence of Highly Virulent Porcine Reproductive and Respiratory Syndrome Virus Variants That Recently Emerged in the United States

**DOI:** 10.1128/genomeA.00772-16

**Published:** 2016-08-04

**Authors:** Aspen M. Workman, Timothy P. L. Smith, Fernando A. Osorio, Hiep L. X. Vu

**Affiliations:** a USDA, ARS, U.S. Meat Animal Research Center, Clay Center, Nebraska, USA; b University of Nebraska–Lincoln, Nebraska Center for Virology and School of Veterinary and Biomedical Sciences, Lincoln, Nebraska, USA

## Abstract

A recent outbreak of particularly virulent disease caused by porcine reproductive and respiratory syndrome virus has occurred in swine herds across the United States. We report here the complete genome sequence of eight viral isolates from four Nebraska herds experiencing an outbreak of severe disease in 2016.

## GENOME ANNOUNCEMENT

Porcine reproductive and respiratory syndrome virus (PRRSV) is an enveloped, positive-sense, single-stranded RNA virus that belongs to the family *Arteriviridae* in the order *Nidovirales* (1). Infection by PRRSV causes reproductive failure in sows and respiratory disease in young pigs (2, 3). Based on genetic and antigenic differences, PRRSV has been divided into two major genotypes: the European genotype (type 1) and the North American genotype (type 2) (4–6). Substantial genetic diversity exists both between and within genotypes, leading to a wide degree of clinical severity ranging from a lack of clinical signs to fatal disease (7).

Since 2014, there have been frequent outbreaks of unusually severe PRRS in the United States. The associated PRRSV isolates have been classified as type 1-7-4 according to a widely used approach for genetic classification based on restriction fragment length polymorphism (RFLP) analysis of the open reading frame 5 (ORF5) (8, 9). However, genetic relatedness/diversity between strains is not adequately described by this approach (10). Therefore, the objective of this work was to obtain the full genome sequence of PRRSV isolates associated with severe disease outbreaks to better understand the molecular characteristics of these newly emerging PRRSV variants.

Serum was collected from infected pigs on four Nebraska farms experiencing virulent PRRSV outbreaks in 2016. From these samples, eight PRRSV variants were isolated using porcine alveolar macrophages (PAMs). Total RNA was purified from the first passage viral supernatant of infected cell cultures using Trizol LS (Life Technologies, Carlsbad, CA). Sequencing libraries were prepared using the Illumina TruSeq RNA Kit and sequenced with 2 × 300 paired end reads on the MiSeq platform (Illumina, San Diego, CA). Index adapters were removed from raw sequence reads using cutadapt (11) and trimmed reads were screened against the UniVec_Core database (NCBI) to remove contaminating vector sequences. Assembly of viral genomes was performed using template-assisted assembly, where trimmed reads were mapped to reference PRRSV genomes (MN184C accession no. EF488739 and NVSL 97-7,895 accession no. AY545985) using Geneious software (version 9.1.3, Biomatters, Auckland, New Zealand [12]). Reads that mapped to the reference genomes were then *de novo* assembled and annotated for each sample (Table 1).

**TABLE 1:** *De novo* genome assemblies of eight virulent PRRSV isolates^a^

PRRSV isolate	Nebraska farm	Genome length (nt)	Mean coverage	RFLP	Accession no.
NCV-13	1	15,097	18,535	1-7-4	KX192112
NCV-16	2	15,101	13,659	1-7-4	KX192113
NCV-17	2	15,097	20,128	1-7-4	KX192114
NCV-21	3	15,098	12,327	1-7-2	KX192115
NCV-23	4	15,107	7,748	1-7-4	KX192116
NCV-24	4	15,097	9,685	1-7-4	KX192117
NCV-25	4	15,098	16,194	1-7-4	KX192118
NCV-26	4	15,097	6,160	1-7-4	KX192119

^a^ nt, nucleotides; RFLP, restriction fragment length polymorphism (8, 9).

The complete genome sequences share 99% nucleotide identity, although *in silico* RFLP analysis of the ORF5 sequence revealed predicted variation. Seven of the eight isolates sequenced here are predicted RFLP type 1-7-4; while the eighth isolate is type 1-7-2 as a result of a single nucleotide substitution in the second SacII restriction site (Table 1). Phylogenetic analysis of PRRSV whole genome sequences reveals that this strain is clearly related to the other 1-7-4 viruses in this study, despite the difference in RFLP classification (not shown). Together, this set of complete genome sequences will further our understanding of PRRSV evolution and provide valuable information to more finely delineate the viral genomic sites associated with changes in viral virulence.

### Accession number(s).

The sequences are deposited in DDBJ/ENA/GenBank under the accession numbers listed in Table 1.
